# High strength chemical peels—not to be tried at home

**DOI:** 10.1002/hsr2.491

**Published:** 2022-01-24

**Authors:** Lisa Killion, Muireann Roche

**Affiliations:** ^1^ Department of Dermatology Beaumont Hospital Dublin Ireland

Since the Covid‐19 pandemic began in January 2020, medical professionals worldwide have had to alter their clinical practice. Many have limited their face‐to‐face consultations and have implemented a teledermatology approach to their mainstream practice. Access to medical care has been limited, and consequently, some patients have tried to replicate professional treatments at home.

A 36‐year‐old woman was referred to the dermatology team with a 2 day history of generalized facial swelling and erythema with acute onset shortness of breath after applying an at‐home chemical peel 2 days prior. Upon arrival to the emergency department, she was treated for severe anaphylaxis in the resuscitation area. She received 500 mcg of intramuscular adrenaline, 200 mg of hydrocortisone, and 10 mg of chlorphenamine intravenously. When stable she reported applying an online purchase of 70% trichloroacetic acid (TCA) to her entire face 2 days prior. Through detailed history taking, no other potential causative factors were found for her anaphylactic reaction and she had no prior history of urticaria, angioedema, or anaphylaxis. She had previously only used TCA peels under the care of a medical professional, with no adverse events.

Once applied, she reported severe stinging and an immediate reaction to her skin described as frosting. Despite immediately washing off the solution, hours later striking facial erythema, edema, and discomfort developed. On examination, she had diffuse facial erythema and edema, with hyperpigmented linear areas of demarcation associated with perioral and periorbital desquamation (Figures 1 and 2). The constellation of clinical findings was consistent with a chemical burn post TCA application. She was commenced on 20 mg of oral prednisolone tapering by 5 mg every 5 days with topical clobetasol propionate ointment and paraffin gel. She was monitored as an inpatient for 48 hours post anaphylaxis and advised to use high sun protection when exposed to daylight once discharged

**FIGURE 1:**
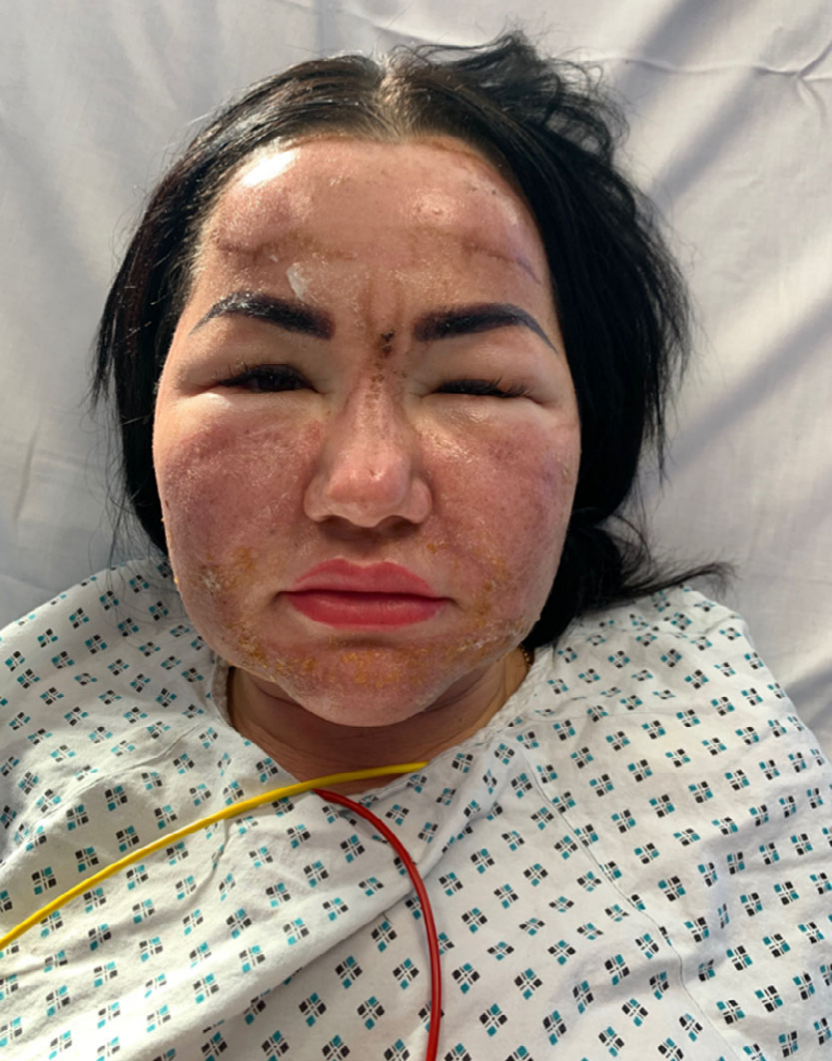
Severe angioedema with background diffuse facial erythema post TCA application

**FIGURE 2:**
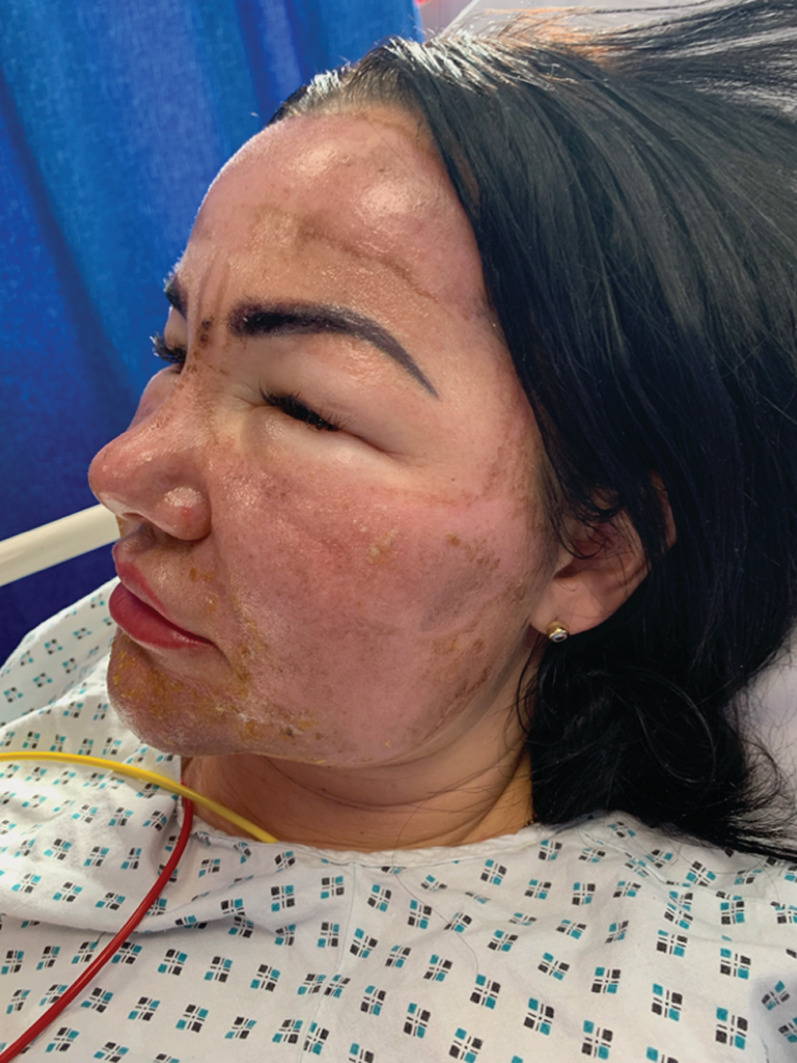
Linear hyperpigmented areas noted on forehead with evident desquamation in a periorbital and oral distribution consistent with that of a chemical burn

We report the first case to our knowledge of an anaphylactic reaction following 70% TCA application. This clinical scenario emphasizes the importance of patient education regarding the adverse effects of peeling agents, as well as the devastating outcomes which can result from misuse. Physicians must be aware of the accessibility of these chemicals outside of the medical setting and the potential of severe anaphylactic reactions when misused.

## FUNDING

No funding sources to declare.

## CONFLICT OF INTEREST

No conflict of interests to declare.

## AUTHOR CONTRIBUTIONS

Conceptualization: Lisa Killion.

Critical Review: Muireann Roche.

Design: Lisa Killion.

Formal Analysis: Lisa Killion, Muireann Roche.

Supervision: Muireann Roche.

Visualization: Lisa Killion.

Writing‐Review and Editing: Lisa Killion, Muireann Roche.

Writing‐Original Draft: Lisa Killion.

All Authors have read and approved the final version of the manuscript. Any additional data can be requested from the main author. Lisa Killion accepts full responsibility for the accuracy and integrity of the data provided.

## TRANSPARENCY STATEMENT

We can confirm that this manuscript is an honest, accurate, and transparent account of the case being reported and that no important aspects of the case have been omitted.

## ETHICS STATEMENT

Ethics approval not required. Patient consent obtained for facial images to be published as displayed.

## Data Availability

Additional data available at request.

